# Meta-genome assembled genome of *Agrobacterium oryzihabitans* associated with the cultivated yellow-green alga *Vaucheria bursata*

**DOI:** 10.1128/mra.00473-25

**Published:** 2026-07-10

**Authors:** Gerardo Laureano, Vignesh Lal, Laurel Mitchell, Elizabeth Santillan Olea, Joseph Tovar, Ashley Scoles, Alok Arun

**Affiliations:** 1 Inter American University of Puerto Rico 19851 , Barranquitas, Puerto Rico, USA; 2 Department of Biological Sciences, California State University Stanislaus 14674, Turlock, California, USA; University of Strathclyde, Glasgow, United Kingdom

**Keywords:** algae, microbiome

## Abstract

We report a draft metagenome-assembled genome (MAG) of an *Agrobacterium* species from *Vaucheria bursata*. The MAG is 89% complete (CheckM2 v1.1.0) with 3,281 predicted genes, providing a basis to explore bacteria–algae interactions and their role in the *Vaucheria* microbiome.

## ANNOUNCEMENT

The exploration of metagenome-assembled genomes (MAGs) provides a robust approach to identifying new taxa from uncultivable prokaryotes. Macroalgae are known to play essential roles in shaping microbial communities through their biological and chemical interactions (1). Species of green algae *Caulerpa* harbor intracellular bacterial endophytes within their rhizoids, which 16S rRNA sequencing revealed to be members of the *Agrobacterium*/*Rhizobium* group closely related to *Agrobacterium tumefaciens* (2). This shows how macroalgae interact with and support beneficial microbes.

In our current study, a novel metagenome-assembled genome (MAG) of *Agrobacterium oryzihabitans* was obtained and assembled from the genomic DNA sequencing data of the freshwater alga *Vaucheria bursata*. Our study supports an earlier finding suggesting the presence of endophytic bacteria in *Vaucheria* sp*.* (3, 4).

Cultures of *Vaucheria bursata* vegetative filaments were obtained from the UTEX culture collection (accession UTEX LB 2067), UT-Austin (Austin, USA). Filaments were grown in 3N Bold Modified medium obtained from UTEX culture collection for 2 weeks under a 16L:8D photoperiod at 25°C. Total genomic DNA was extracted from filaments using the NucleoSpin Plant II Kit (Macherey-Nagel) following the manufacturer’s instructions. DNA libraries were prepared using the NEBNext Ultra II DNA Library Prep Kit from Illumina. Genomic DNA (500 ng) was fragmented to ~350 bp, end-repaired, A-tailed, and ligated to indexed adapters. Libraries were purified, PCR-amplified (five to eight cycles), assessed for quality on a Bioanalyzer, and sequenced on an Illumina NovaSeq with paired-end 150 bp reads. The sequencing yielded a total of 335,501,104 reads. All software was run using default parameters unless otherwise specified. Low-quality read trimming and quality verification were performed using Fastp v0.23.4 (5). The MetaWrapv1.3 pipeline was applied for MAG recovery, resulting in a total of 11 recovered MAGS (6). Among the recovered MAGs, MAG_3 was selected for further analysis in this study. To assign a taxonomic identity, we analyzed the genome using GTDB-tk v2.4.0, which classified the MAG at the species level as *Agrobacterium oryzihabitans* (7). The genome quality was evaluated using CheckM2 v1.1.0, which estimated 89.8% completeness and 0.78% contamination (8). A BUSCO v5.8.3 assessment using the *Rhizobium/Agrobacterium* lineage further indicated a genome completeness of 80.7% (single-copy BUSCOs: 80.69%; duplicated: 0.1%) (9).

**Fig 1:**
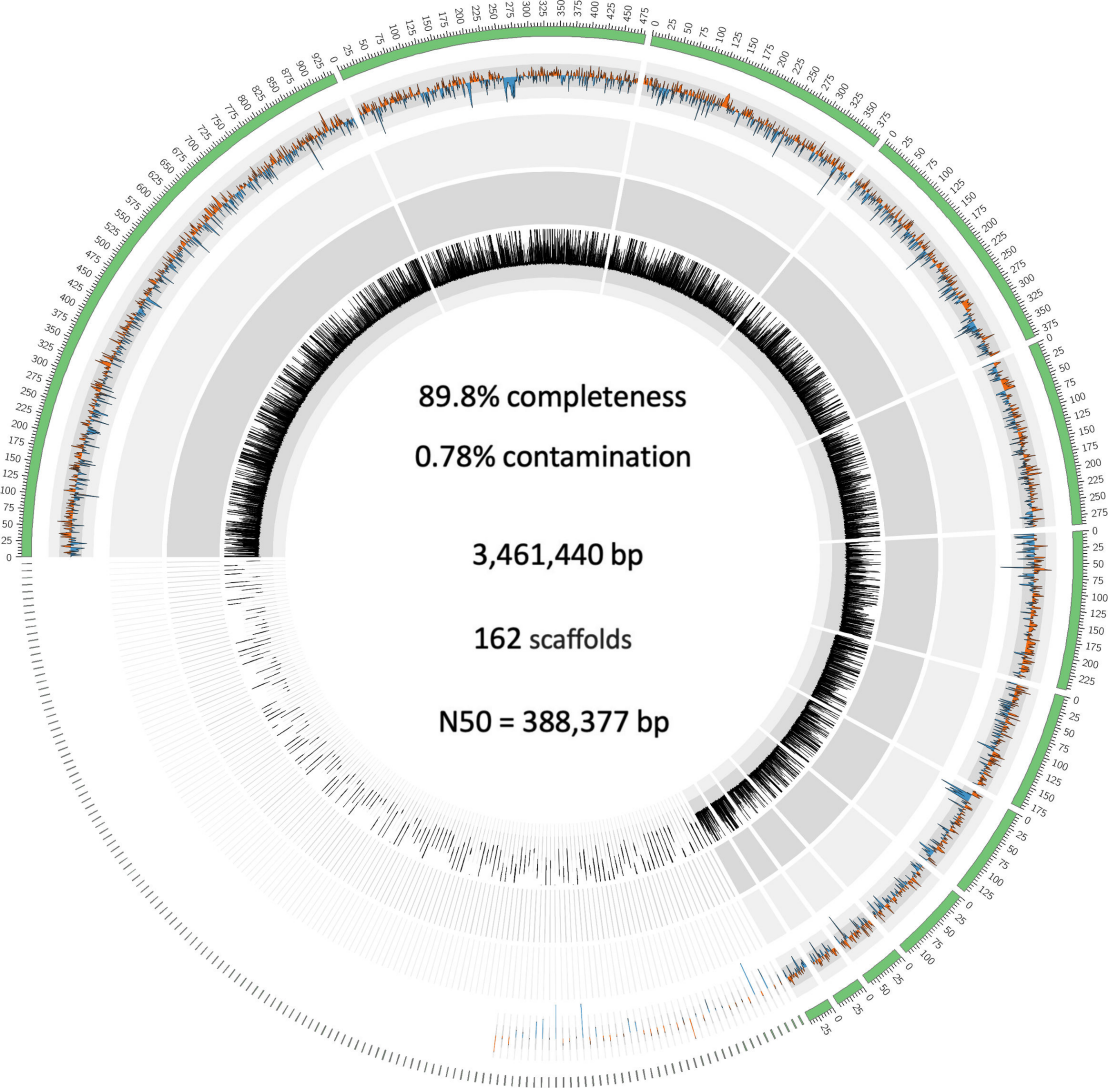
Circos plot of the *Agrobacterium oryzihabitans* metagenome-assembled genome (MAG). Green bars represent contigs, and the red bar denotes assembly gaps. Bar thickness corresponds to contig length in kilobases. GC content is shown in 1,000 bp windows relative to the MAG-wide mean. The innermost ring displays the percent deviation in coverage for each 1,000 bp window compared to the average MAG coverage. The plot was generated using Koonkie Inc. *circos_mag* v1.0.0 (https://github.com/Koonkie-Cloud-Services/circos_mag).

The *Agrobacterium oryzihabitans* MAG showed a total gapped genome length of 3,461,440 bp (N50 = 388,377 bp) distributed across 186 contigs and arranged in 162 scaffolds, with a guanine and cytosine (GC) content of 59.40% (Fig. 1). Gene prediction and annotation were performed using Prokka v1.14.6 (version 1.14.6 + galaxy1) (10). A total of 3,281 genes were functionally annotated with putative functions (Table 1). The total number of reads (2,693,312) associated with the *Agrobacterium oryzihabitans* MAG was deposited to National Center for Biotechnology Information (NCBI) Sequence Read Archive (SRA) (SRX27942632).

**TABLE 1:** Genome features of the *Agrobacterium oryzihabitans* MAG

Parameters	Statistics
Gapped genome length (bp)	3,461,440
Number of scaffolds	162
N50 (bp)	388,377
GC (%)	59.40
Predicted CDS	3,281

The availability of this MAG, together with other bacteria previously reported from *Vaucheria bursata* (3), provides an opportunity to investigate the potential metabolic capacities and interactions between these MAGs and the macroalga, offering deeper insight into the complex microbiome of *V. bursata*.

## Data Availability

MAG was deposited at GenBank under accession number JBNGFZ000000000.1. The raw sequencing reads of the MAG are available with accession number SRR32638075 and deposited in BioProject PRJNA1020787. All annotation data utilized in this paper are deposited in a Zenodo repository, https://doi.org/10.5281/zenodo.15801375, and raw reads can be accessed in NCBI SRA database (SRX30956066). The genome is deposited in NCBI under the name *Rhizobium oryzihabitans*. However, according to the GTDB taxonomy framework, this organism is assigned to a different taxonomic placement, and our interpretation follows the GTDB classification.
